# *A Heavy Feeling in the Stomach*: Neural Correlates of Anxiety in Crohn's Disease

**DOI:** 10.1111/nmo.70029

**Published:** 2025-03-24

**Authors:** Silvia Tempia Valenta, Sara Ventura, Francesca Benuzzi, Fernando Rizzello, Paolo Gionchetti, Diana De Ronchi, Anna Rita Atti, Alessandro Agostini, Nicola Filippini

**Affiliations:** ^1^ Department of Biomedical and Neuromotor Sciences University of Bologna Bologna Italy; ^2^ Doctoral Program of Global Health, Humanitarian Aid and Disaster Medicine Vrije Universiteit Brussel Bruxelles Belgium; ^3^ Department of Clinical and Surgical Sciences IRCCS Sant'Orsola‐Malpighi Hospital, University of Bologna Bologna Italy; ^4^ Department of Biomedical, Metabolic and Neural Sciences University of Modena and Reggio Emilia Modena Italy; ^5^ IRCCS San Camillo Hospital Venice Italy

**Keywords:** anxiety, Crohn's disease, fMRI, functional connectivity, visceral sensitivity

## Abstract

**Introduction:**

Crohn's disease (CD) is a chronic inflammatory condition associated with psychological stress and anxiety. Functional magnetic resonance imaging (fMRI) studies have shown differences in brain function between patients with CD and healthy controls (HC). This study aimed to compare the neural correlates of anxiety inindividuals with CD relative to HC, using resting‐state fMRI data.

**Methods:**

Participants filled in the State–Trait Anxiety Inventory (STAI), a validated tool for measuring anxiety, and underwent an MRI acquisition, including both structural and functional sequences, to identify brain regions associated with anxiety scores.

**Results:**

Seventeen patients with CD and eighteen HC matched for age, education, and sex participated in the study. No significant group differences emerged in the STAI scores. However, resting‐state fMRI analysis revealed distinct patterns of functional connectivity associated with anxiety scores for the two study groups. Among CD group, greater STAI scores correlated with increased functional connectivity, whereas, in HC, they correlated with decreased functional connectivity. Significant clusters were found in brain regions belonging to specific resting‐state networks (RSNs): (a) Posterior Cingulate Cortex (PCC, within the Default Mode Network), (b) left Middle Frontal Gyrus (within the Left Fronto‐Parietal Network), and (c) PCC and right Superior Temporal Gyrus (within the Dorsal Attention Network).

**Conclusion:**

The differential association between functional connectivity and STAI scores observed for CD and HC participants was located in areas within self‐referential (Default Mode Network) and cognitive (Left Fronto‐Parietal Network and Dorsal Attention Network) RSNs. Our findings suggest that maladaptive/dysfunctional processing of negative emotions and visceral sensitivity may occur in patients with CD.

## Graphical abstract

Resting‐state fMRI revealed distinct anxiety‐related functional connectivity patterns in Crohn's disease (CD) patients and healthy controls. In CD, higher anxiety correlated with increased connectivity, while in controls, it correlated with decreased connectivity. Findings suggest altered neural processing of emotions and visceral sensitivity in CD, involving self‐referential and cognitive networks.

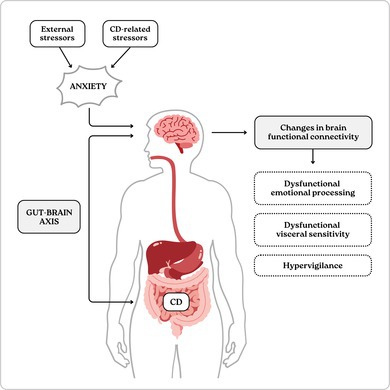

**Summary**

- Patients with Crohn's disease (CD) show distinct functional connectivity patterns linked to anxiety scores in self‐referential (Default Mode Network) and cognitive (Left Fronto‐Parietal Network and Dorsal Attention Network) resting‐state networks.
- Significant clusters were specifically located in the Posterior Cingulate Cortex, the left Middle Frontal Gyrus, and the right Superior Temporal Gyrus.
- Our findings may indicate a maladaptive or dysfunctional processing of negative emotions and visceral sensitivity in individuals with CD.

## 1 Introduction

Crohn's disease (CD) is an inflammatory bowel disease (IBD) affecting around 0.2% of the Western population [1, 2]. CD typically arises between the ages of 20 and 30 and is one of the most common chronic conditions affecting young adults [3]. CD has a multifactorial etiology that includes immune‐mediated components [4, 5]. The clinical course of the disease is marked by periods of remission and flare‐ups, during which individuals experience symptoms such as abdominal pain, diarrhea, and rectal bleeding [6]. Additionally, extra‐intestinal manifestations may occur, including fever, fatigue, joint pain, skin lesions, eye inflammation, and liver complications [7]. In the long term, patients can have weight loss, strictures, fistulas, malabsorption, and nutritional deficiencies [6, 8]. Modern treatment for CD focuses not only on clinical remission but also on endoscopic healing and histological improvement [9].

Despite treatment advancements, CD still leads to a significant psychological burden on affected individuals [10, 11, 12, 13]. Psychiatric comorbidity is common in patients with IBD compared to the general population, as shown by recent large population‐based studies involving thousands of individuals [14, 15]. The incidence rate ratios (IRRs) for depression, anxiety, bipolar disorder, and schizophrenia were found to be significantly higher in IBD patients relative to controls, with IRRs of 1.58, 1.39, 1.82, and 1.64, respectively [15]. These rates were consistent for both CD and ulcerative colitis (UC), the most common types of IBD. More recent literature also revealed an increased prevalence of post‐traumatic stress and disordered eating behaviors in CD patients relative to controls [16]. Furthermore, psychiatric comorbidities represent a significant aggravating factor causing both a decline in the quality of life [17] and poorer treatment outcomes, with higher rates of surgery and increased mortality [15] among patients with CD.

Anxiety in patients with CD is fueled by the constant threat of disease relapse or comorbidity onset [18]. This state of anticipation and uncertainty can, in turn, exacerbate anxiety and trigger further relapses, given the well‐known association between stress and autoimmunity [18]. Up to a third of patients experience anxiety symptoms, with the prevalence rising to 50% in those with active disease [19]. The unpredictability of the disease's course severely impacts the individual's functioning and autonomy [20, 21]. Patients may experience feelings of isolation and withdrawal from social and professional activities, heightening the emotional and psychological distress associated with the disease [22, 23]. Furthermore, the experience of anxiety in individuals with CD can be influenced by visceral sensitivity to gut‐related sensations, which can lead to disproportionate emotional responses [24]. This form of anxiety is not only driven by external stressors, but it is also tied to the individual's altered perception of their body and its functioning [25, 26]. This triggers hypervigilance and hyperarousal mechanisms related to gastrointestinal functioning, with possible misinterpretation of physiological or para‐physiological signals such as borborygmi, belching, visible peristalsis, or gastroesophageal reflux.

Brain imaging studies, using functional Magnetic Resonance Imaging (fMRI) technique, either task‐based or at rest, have consistently shown alterations in brain activity [27, 28, 29] and increased functional connectivity [30, 31, 32] in patients with CD, in regions associated with pain processing, emotional regulation, and cognitive functions [32, 33]. A study by Rubio et al. found that CD patients had greater brain activity relative to controls in areas such as the insula, the amygdala, and the thalamus, which are known to be related to interoceptive awareness, emotional responses, sensory relay, and pain processing [18]. Moreover, the reported increased brain activity was found to correlate with measures such as gastrointestinal symptom‐specific anxiety, uncertainty about visceral sensations, and intolerance of uncertainty [18]. A very recent review reported that the Default Mode Network, Central Executive Network, and Limbic Network were particularly sensitive to differentiating active and inactive CD states or CD patients and controls [34]. However, while previous research has demonstrated alterations in brain activity and functional connectivity in patients with CD at different stages of the disease and/or relative to healthy controls (HC), the specific role of anxiety in modulating resting brain networks remains scarcely investigated. This study aims to compare the neural mechanisms of anxiety in patients with CD and HC by analyzing functional connectivity in brain regions linked to emotional regulation and visceral sensitivity using resting‐state fMRI. Based on previous studies, we hypothesize that differential association between resting state measures and anxiety scores in patients with CD and HC will be located in resting state networks associated with cognitive, emotional, and/or self‐referential processing rather than networks mostly associated with sensorial aspects.

## 2 Materials and Method

### 2.1 Study Design and Participants

The study was a prospective, single‐center observational investigation conducted in Emilia Romagna, Italy. We included individuals with CD and HC, all of whom provided informed consent to the processing of their personal data. Inclusion criteria were age between 18 and 50, diagnosis of CD at least 3 years prior to enrollment, and being right‐handed. Exclusion criteria were claustrophobia and the presence of metallic implants, a previous history of neurological or psychiatric conditions including psychotic, anxiety, depressive, and bipolar disorders, as well as substance use, eating, and personality disorders, the use of psychotropic medications in the past 12 months, corticosteroid use in the last 6 months, pregnancy, and planned surgery. Participants having a first‐degree relative diagnosed with CD were excluded to minimize potential confounding factors related to genetic or familial predisposition. HC participants were recruited from the University of Bologna staff. Patients with CD were recruited at the IBD Unit of Sant'Orsola‐Malpighi University Hospital in Bologna, which is the Italian referral center for the research and care of patients with IBD.

The study was approved by the local Ethics Committee (Prot. n 2453/ce N. di pratica ce 45/14), Comitato Etico Provinciale di Modena Azienda Ospedaliero‐Universitaria. The research was conducted in accordance with the ethical principles of the Declaration of Helsinki and Good Clinical Practice guidelines.

### 2.2 Procedures and Measures

Socio‐demographic data, including age, gender, and education, were collected during the recruitment stage for both study groups. According to the Montreal Classification [35], fully trained physicians of the IBD Unit collected the following data for patients with CD: age at diagnosis, disease location, disease behavior, perianal disease, extra‐intestinal manifestations, history of intestinal surgery, biologic treatment, and current maintenance therapies. Endoscopic data were obtained from routine ileocolonoscopies that were conducted no more than 2 weeks before the MRI scan. The endoscopic activity was defined based on the simple endoscopic score for CD (SES‐CD). The clinical activity was defined based on the Crohn's Disease Activity Index (CDAI) [36]. Clinicians of the IBD Unit classified CD patients as follows: (A) Remission: CDAI < 150, SES‐CD < 3; (B) Mild Activity: CDAI between 150 and 250, or SES‐CD between 3 and 6; (C) Moderate Activity: CDAI > 250, or SES‐CD between 7 and 15.

In addition, the clinical assessment included a screening for neurological and psychiatric disorders based on the Mini International Neuropsychiatric Interview (MINI), a diagnostic tool based on the Diagnostic and Statistical Manual of Mental Disorders, Fourth Edition (DSM‐IV) [37]. The MINI is a structured questionnaire designed to assess DSM‐IV psychiatric conditions, allowing for reliable and consistent diagnoses. Subsequently, all participants filled in the State–Trait Anxiety Inventory (STAI‐Y) [38], a self‐report questionnaire assessing anxiety levels. The questionnaire includes 40 items, with 20 items dedicated to evaluating state anxiety (the temporary feeling of anxiety in response to a situation) and 20 items assessing trait anxiety (the general tendency to experience anxiety). The STAI‐Y helps to determine both the presence and severity of anxiety symptoms, as well as the individual's overall predisposition to anxiety. In this study we specifically used the STAI state subscale, and all references to STAI scores throughout this paper pertain exclusively to this subscale.

HC participants underwent the same screening procedures, including neuropsychological assessment and MRI protocol as the patients with CD, with the exception of CD‐specific clinical examinations.

### 2.3 MRI Data Acquisition and Preprocessing

#### 2.3.1 MRI Data Acquisition

Neuroimaging data acquisition was performed at the Ospedale Civile di Baggiovara in Modena, Italy. A Philips Intera system at 3.0 Tesla equipped with an eight‐channel head coil was used. The neuroimaging protocol used in the present study included both functional and structural imaging sequences.

For the structural MRI acquisition, 3D high‐resolution T1‐weighted MR images were acquired using an MPRAGE sequence (TR = 9.9 ms, TE = 4.6 ms, 170 sagittal slices, voxel dimension = 1 mm isotropic).

Resting‐state functional MRI (rs‐fMRI) whole‐brain functional imaging data was acquired using a gradient echo EPI sequence (TR = 2000 ms, TE = 35 ms, field of view = 240 mm, voxel dimension = 3 × 3 × 4 mm, number of volumes = 240). For the rs‐fMRI scan, participants were instructed to lie still in a dimly lit environment, keeping their eyes open, then to avoid focusing on any specific thoughts, and to remain awake.

#### 2.3.2 MRI Data Preprocessing

Both structural MRI and rs‐fMRI data underwent standardized preprocessing and analysis procedures. Imaging preprocessing for structural MRI included: (1) reorienting the images to the standard template (MNI), (2) applying bias field correction, (3) performing brain extraction, and (4) segmenting brain tissues into (a) gray matter (GM), (b) white matter (WM), and (c) cerebrospinal fluid (CSF) using FMRIB's Automated Segmentation Tool (FAST). This process provided measures of total GM, WM, and CSF volumes for each of the study participants. A voxel‐based morphometry (VBM) approach was used with FSL‐VBM [39] using default settings. Brain extraction and tissue‐type segmentation were performed, and resulting GM partial volume images were aligned to the standard MNI template using registration tools both linear (FLIRT) and nonlinear (FNIRT). Then, images were averaged, modulated (by the warp field Jacobian), and smoothed with an isotropic Gaussian of 6 mm full‐width at half max (FWHM). This approach was used in order to (a) investigate any potential GM difference between CD and HC groups and (b) identify any potential differential association between GM volume and STAI scores for the two study groups.

The rs‐fMRI data were analyzed using the fMRI Expert Analysis Tool (FEAT) v6.00 [40]. The individual pre‐statistical preprocessing steps consisted of motion correction, brain extraction, spatial smoothing using a Gaussian of FWHM (full width at half maximum) 5 mm, and high‐pass temporal filtering with a cut‐off of 100 s (0.01 Hz). Then, functional volumes were aligned to each participant's structural MRI and normalized to standard space using the FLIRT and FNIRT registration tools, and finally optimized using a boundary‐based registration approach [41]. Given the susceptibility of rs‐fMRI signals to the presence of artifacts that may spatially or spectrally overlap with resting‐state networks (RSNs), we applied the FIX tool [42] to denoise functional images. Denoised and preprocessed functional data, comprising 240 time points per subject, were temporally concatenated across the subjects to create a unified 4D dataset and derive our population‐based RSNs. This was achieved using a tool called Multivariate Exploratory Linear Optimized Decomposition into Independent Components (MELODIC) [43]. The number of components was fixed at 25 based on initial population modeling, which determined that only 25 components were significantly non‐zero on average. RSNs of interest covered the entire brain and were selected by spatially correlating the data with predefined reference maps (https://www.fmrib.ox.ac.uk/datasets/brainmap+rsns/). Group‐level comparisons were performed using a dual regression approach [44], that is, a regression‐based technique facilitating voxel‐wise analysis of functional connectivity maps, allowing for detailed examination of group differences in resting‐state brain connectivity.

### 2.4 Statistical Analyses

Statistical analysis was performed using SPSS software (v 25.0) and MRI data were processed using the FMRIB Software Library (FSL, www.fmrib.ox.ac.uk/fsl) (Smith et al. 2004).

Group comparisons of continuous variables, including socio‐demographic factors and STAI scores, were conducted using *T*‐tests, whereas the Chi‐square (*χ* ^2^) test was used to examine differences in the binary variable (sex). These preliminary analyses were performed to ensure that any socio‐demographic or baseline differences between the patients with CD and HC were identified and accounted for.

With regard to the imaging data, the scores derived from the STAI questionnaire were included as separate covariates of interest for the general linear model (GLM) analyses on structural and resting‐state fMRI data. The objective was to identify those brain regions reflecting group differences (i.e., differences in “correlation slopes”) associated with the STAI state scores. Voxel‐wise GLM was applied on GM and RSN maps using randomize, a permutation‐based nonparametric testing (5000 permutations) [45], and threshold‐free‐cluster‐enhancement (TFCE) for cluster identification [46]. Family Wise Error (FWE) corrected cluster significance threshold of *p* < 0.05 and number of voxels exceeding 50 were applied to the suprathreshold clusters.

## 3 Results

### 3.1 Sample Sociodemographic, Psychometric, Neuroanatomical, and Clinical Characteristics

The study included 17 patients with CD and 18 HC. There were no significant differences between the two groups in terms of age, sex, or years of education. Similarly, no group‐related differences were observed in the STAI scores. Moreover, no significant group‐related differences were observed in brain structural measures, including total intracranial volume, gray matter, white matter, and cerebrospinal fluid volume. For details, please refer to Table 1.

**TABLE 1:** Socio‐demographic, psychometric, and neuroanatomical characteristics of the two study groups. Numbers reflect mean (± standard deviation), or number of subjects.

	Controls, *N* = 18	Patients with CD, *N* = 17	*p*‐value
Socio‐demographics			
Age (Y)	28.33 (± 5.47)	30.12 (± 5.96)	0.36
Education (Y)	16.06 (± 2.51)	15.24 (± 2.73)	0.36
Sex (M/F)	8/10	6/11	0.58
Disease Duration (Y)	NA	10 (± 5.66)	
STAI questionnaire			
State Anxiety	32.06 (± 4.26)	33.88 (± 6.76)	0.34
Brain features			
Total Intracranial Volume, cc	1392.4 (± 109.6)	1340.8 (± 155.1)	0.26
Total Gray Matter*	41.19 (± 1.68)	41.21 (± 1.74)	0.97
Total White Matter*	36.14 (± 1.41)	35.84 (± 1.45)	0.54
Total Cerebrospinalfluid*	22.66 (± 0.95)	22.94 (± 1.72)	0.55

Abbreviations: CD = Crohn Disease; M/F = male/female; NA = not applicable; Y = years.

* Values are expressed as a percentage of total intracranial volume.

Using the Montreal Classification, the enrolled patients were categorized as follows. Age at diagnosis: 2 patients were in A1 (age at diagnosis < 16 years), and 15 in A2 (age at diagnosis between 16 and 40 years). Disease location (L): 7 patients had ileal involvement (L1), and 10 had ileocolonic involvement (L3). Disease behavior (B): 11 patients had non‐stricturing and non‐penetrating disease (B1), [2 of them were classified as B1p (non‐stricturing/non‐penetrating with perianal disease)], 3 patients had stricturing (B2), and 3 patients had penetrating (B3) disease. Finally, 2 patients had penetrating disease with perianal disease (B3p). Using the CDAI, 14 patients were classified in remission because of a CDAI score < 150, whereas 3 had mild disease activity with a CDAI score ranging between 150 and 250. Moreover, using the SES‐CD score, 14 patients were defined as being in endoscopic remission (SES‐CD < 3) whilst 3 patients had mild endoscopic activity (SES‐CD between 3 and 6). As for extraintestinal manifestations, 6 patients had arthralgia, and 1 patient had erythema nodosum. Six patients had previously undergone IBD‐related surgeries, and 1 patient had an ostomy. Current treatments of the enrolled CD patients were: biologics (infliximab, adalimumab) for 6 patients, azathioprine for 3, and 5‐aminosalicylic acid (5ASA) for 8.

### 3.2 Imaging Data Analyses

Voxel‐wise GLM analysis of structural MRI data did not reveal any significant differences between the two study groups. Moreover, when investigating the relationship between brain morphological measures and the STAI score, we did not observe any differences between the two groups.

For the rs‐fMRI data, a trained neuroscientist (NF) visually inspected data preprocessing in order to ensure accurate registration. The optimal threshold for FIX was determined to be 30, with a median true positive rate (TPR) of 95.3% (95.8%) and a true negative rate (TNR) of 90.7% (92.5%), exceeding the recommended thresholds that are TPR > 95% and TNR > 70%.

**FIGURE 1:**
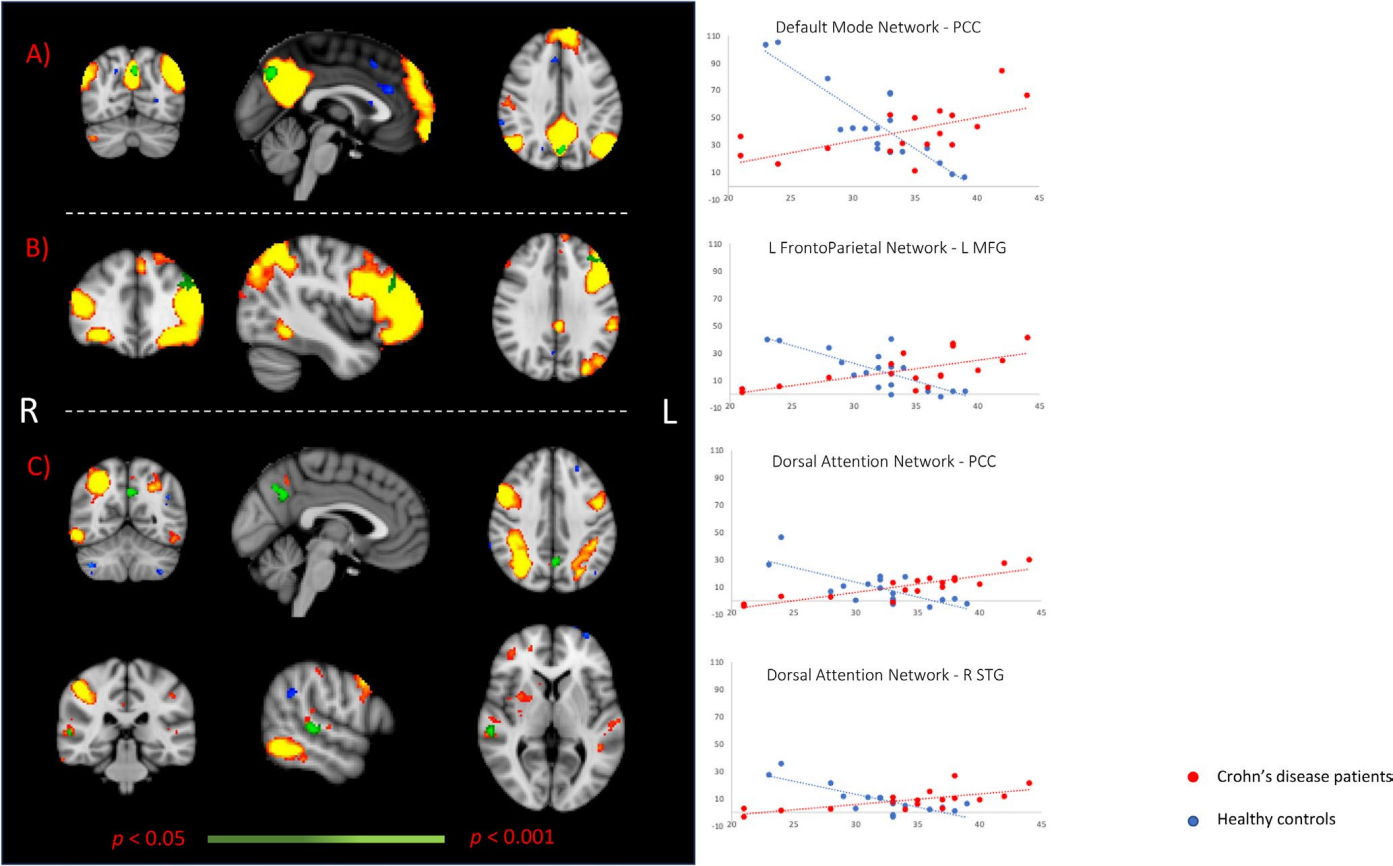
The image depicts differences between Crohn's disease (CD) patients and healthy controls (HC) in voxel‐wise correlations between the State–Trait Anxiety Inventory (STAI) score and functional connectivity measures within the Default Mode Network (A), the left Fronto‐Parietal Network (B) and the Dorsal Attention Network (C). Significant clusters are shown in green overlaid on the respective resting‐state network.

A group‐related differential association between the STAI score and functional connectivity measures was observed for three RSNs, namely the Default Mode Network (DMN), the Left Fronto‐Parietal Network, and the Dorsal Attention Network. Specifically, for all three RSNs, increased functional connectivity was observed in CD group with higher scores on the STAI test compared to HC, who exhibited the opposite trend. In detail, within the DMN, this effect was found in the Posterior Cingulate Cortex (PCC) [reporting here and below, cluster size in voxels, T‐Max and peak coordinates in standard MNI space: 97, 6.85, (−2, −70, 38)] (Figure 1A). Within the Left Fronto‐Parietal Network, the significant difference was observed in the left Middle Frontal Gyrus [64, 5.45, (−36, 38, 32)] (Figure 1B). Finally, two significant clusters were identified within the Dorsal Attention Network, one located in the PCC [105, 6.96 (−2, −58, 36)] and one in the right Superior Temporal Gyrus [52, 6.54 (58, −28, 2)] (Figure 1C). No group‐related differences were observed when the DMN, Left Fronto‐Parietal Network, and the DAN were compared between the two study groups, not taking into consideration the STAI scores.

## 4 Discussion

Resting‐state fMRI analysis revealed distinct patterns of functional connectivity associated with state anxiety scores in patients with CD and a group of HC. In the CD group, higher STAI scores were linked to increased connectivity, whereas, in HC, higher STAI scores were correlated with decreased functional connectivity. Significant clusters were identified in three separate resting‐state networks (RSNs)—Default Mode Network (DMN), Left Fronto‐Parietal Network, and Dorsal Attention Network—and were specifically located in the Posterior Cingulate Cortex (PCC), the left Middle Frontal Gyrus, and the right Superior Temporal Gyrus. No differences were observed in gray matter volume or its differential association with STAI scores between the study groups.

Our findings in HC are in line with previous research on individuals without organic illnesses, which demonstrate reduced functional connectivity in functional brain networks associated with anxiety and stress [47]. Consistently, this pattern was observed in studies measuring anxiety with STAI scores [48, 49, 50, 51]. In particular, a recent meta‐analysis showed that anxiety is characterized by altered intrinsic connectivity, including hypo‐connectivity between affective, executive control, and default mode networks, reduced connectivity between salience and sensorimotor networks, and attenuated anti‐correlations between executive control and default mode networks [52].

In the pathological context of CD, our study was the first to identify significant clusters of increased connectivity linked to anxiety scores in areas within self‐referential (DMN) and cognitive (Left Fronto‐Parietal Network and Dorsal Attention Network) RSNs. Specifically, we found involvement of the PCC, an area known to be associated with self‐referential thinking [53], the left Middle Frontal Gyrus, a region related to cognitive control and memory [54, 55], and the Superior Temporal Gyrus, an area linked to social cognition, auditory processing, and language comprehension [56]. Furthermore, all these areas have been shown to be involved in emotional regulation processing [53, 57, 58, 59, 60]. A previous study by Thomann et al. 2017 found consistent results, showing increased connectivity in the DMN subsystems, including middle cingulate activity, linked to anxiety scores in patients with CD [61]. In contrast, recent findings have reported decreased functional connectivity in amygdala subregions and the hippocampus, middle frontal gyrus, insula, precuneus, and postcentral gyrus [62], suggesting divergent patterns of connectivity depending on the brain regions and the networks assessed.

Taken together, our findings may indicate a maladaptive/dysfunctional processing of negative emotions, visceral sensitivity, and cognitive processes occurring in patients with CD. In particular, increased functional connectivity in the PCC in this clinical population could be interpreted as a reflection of hypervigilance on internal body states due to a fear of flare‐ups [32]. Regarding the left Middle Frontal Gyrus, higher connectivity in CD patients could suggest higher cognitive and emotional regulation demands relative to HC [63]. Finally, the increased connectivity in the right Superior Temporal Gyrus could indicate higher sensitivity to social and emotional contexts [34]. The increase in connectivity observed in patients with can possibly reflect a hyperactivation of brain networks driven by gut‐brain axis dysfunction [30, 31, 32]. These findings are also in line with psychological research indicating a higher prevalence of anxiety and associated regulatory challenges related to CD [18, 19, 64, 65].

From a clinical perspective, our results may provide the neuronal substrate of the mental health concerns repeatedly reported by patients with CD. Indeed, young chronic patients face the constant burden of symptoms that they may endure for decades, potentially amplifying their sense of vulnerability [66, 67]. It is essential that patients receive the necessary support to develop anxiety self‐regulation strategies, ultimately restoring a sense of control and self‐efficacy over their health concerns [11, 68]. Clinical research carried out over the last decades has shown the efficacy of various psychotherapeutic approaches in improving psychological functioning and reducing anxious‐depressive symptoms in IBD [69, 70]. Specifically, studies have highlighted the benefits of cognitive behavioral therapy (CBT) [71, 72], mindfulness‐based cognitive therapy (MBCT) [73], acceptance and commitment therapy (ACT) [74, 75], and psychological or psychosocial group‐based intervention programs [76]. Institutions such as the European Crohn's and Colitis Organization (ECCO), the American Gastroenterological Association (AGA), and the British Society of Gastroenterology (BSG) emphasize the importance of incorporating psychological assessments and interventions into the management of IBD. However, despite this recognition, psychological support remains insufficient in many countries and regions.

The main limitation of our study was the small sample size, which may have limited the power to detect significant differences for psychological variables and did not allow us to stratify the sample to directly compare patients with CD with different levels of anxiety. Furthermore, the small sample size precluded stratification by disease severity and limited our ability to further investigate potential associations between inflammatory mediators and brain activity. Moreover, here we used a semi‐structured interview to exclude the presence of mood disorders in our participants, but future studies should also include more specific questionnaires to assess the impact of current mood on our results. Furthermore, the inflammatory activity in three patients was mild, and this could have modified the brain activity of the patients through the activation of vagus nerve signals from the intestine to the brain [77] or circumventricular organs. This issue warrants further investigation, and further research should compare groups of patients of suitable size both in remission and in disease activity. Moreover, disease activity should also be assessed by measuring markers such as fecal calprotectin and should not be based merely on clinical and endoscopic indices, as was done in the present study. Finally, here we have used a cross‐sectional design based on a single time point. A longitudinal approach is warranted as it could provide further insights into how the functional connectivity patterns in patients with CD may evolve over time, particularly in response to changes in disease activity, medical treatments, or psychological interventions.

In conclusion, the present study may have enhanced our understanding of the neural correlates of anxiety among patients with CD, showing increased functional connectivity in key resting‐state networks associated with self‐referential and cognitive control processing. These findings hold potential as imaging markers for monitoring disease progression and identifying promising targets for innovative therapeutic interventions, with a particular emphasis on improving the long‐term quality of life for these patients.

For significant clusters, scatterplots are provided to illustrate the relationship between functional connectivity values (*y* axis) and STAI scores (*x* axis), with red dots representing CD patients and blue dots reflecting HC. PCC defines Posterior Cingulate Cortex, L MFG defines Left Middle Frontal Gyrus, R STG defines Right Superior Temporal Gyrus. All images display results at *p* < 0.05, corrected for multiple comparisons (dark‐to‐light green color scale). R indicates the brain's right hemisphere, while L indicates the left.

## Author Contributions

S.T.V.: manuscript writing, S.V.: data management, F.B.: study design and data acquisition, F.R. and P.G.: patient enrollment and evaluation, D.D.R. and A.R.A.: manuscript supervision, A.A.: study concept and design, manuscript writing, N.F.: study design, imaging data analysis, manuscript writing.

## Ethics Statement

The study was approved by the local Ethics Committee (Prot. n 2453/ce N. di pratica ce 45/14), Comitato Etico Provinciale di Modena Azienda Ospedaliero‐Universitaria. All participants provided written informed consent prior to their involvement in the study.

## Consent

Consent for Publication. All authors have provided their consent for publication.

## Conflicts of Interest

The authors declare no conflicts of interest.

## Data Availability

The data that support the findings of this study are available from the corresponding author upon reasonable request.
